# Fentanyl Enhances Hepatotoxicity of Paclitaxel via Inhibition of CYP3A4 and ABCB1 Transport Activity in Mice

**DOI:** 10.1371/journal.pone.0143701

**Published:** 2015-12-03

**Authors:** Jing-Dun Xie, Yang Huang, Dong-Tai Chen, Jia-Hao Pan, Bing-Tian Bi, Kun-Yao Feng, Wan Huang, Wei-An Zeng

**Affiliations:** 1 Department of Anesthesiology, Sun Yat-sen University Cancer Center, State Key Laboratory of Oncology in South China, Collaborative Innovation Center for Cancer Medicine, Guangzhou, Guangdong, China; 2 Department of Clinical Trial Center, Sun Yat-sen University Cancer Center, State Key Laboratory of Oncology in South China, Collaborative Innovation Center for Cancer Medicine, Guangzhou, Guangdong, China; Universidade Federal do Rio de Janeiro, BRAZIL

## Abstract

Fentanyl, a potent opioid analgesic that is used to treat cancer pain, is commonly administered with paclitaxel in advanced tumors. However, the effect of fentanyl on the hepatotoxicity of paclitaxel and its potential mechanism of action is not well studied. The purpose of this study was to investigate the effect of fentanyl on the hepatotoxicity of paclitaxel and its potential mechanisms of action. Pharmacokinetic parameters of paclitaxel were tested using reversed phase high-performance liquid chromatography (RP-HPLC). Aspartate transaminase (AST), alanine aminotransferase (ALT), and mouse liver histopathology were examined. Moreover, the cytotoxicity of anti-carcinogens was examined using 1-(4, 5-dimethylthiazol-2-yl)-3,5-diphenylformazan (MTT), and the intracellular accumulation of doxorubicin and rhodamine 123 was detected by flow cytometry. Furthermore, the expression of ABCB1 and the activity of ABCB1 ATPase and CYP3A4 were also examined. In this study, the co-administration of fentanyl and paclitaxel prolonged the half-life (t_1/2_) of paclitaxel from 1.455 hours to 2.344 hours and decreased the clearance (CL) from 10.997 ml/h to 7.014 ml/h in mice. Fentanyl significantly increased the levels of ALT in mice to 88.2 U/L, which is more than 2-fold higher than the level detected in the control group, and it increased the histological damage in mouse livers. Furthermore, fentanyl enhanced the cytotoxicity of anti-carcinogens that are ABCB1 substrates and increased the accumulation of doxorubicin and rhodamine 123. Additionally, fentanyl stimulated ABCB1 ATPase activity and inhibited CYP3A4 activity in the liver microsomes of mice. Our study indicates that the obvious hepatotoxicity during this co-administration was due to the inhibition of CYP3A4 activity and ABCB1 transport activity. These findings suggested that the accumulation-induced hepatotoxicity of paclitaxel when it is combined with fentanyl should be avoided.

## Introduction

Paclitaxel, a mitotic inhibitor, is widely used in breast cancer, ovarian cancer and non-small cell lung cancer as the first-line or second-line anti-carcinogen in clinical practice[1–3]. Despite encouraging clinical effects, the side effects of paclitaxel, including hepatotoxicity, cardiovascular toxicity and neurotoxicity, cannot be ignored. The liver is the primary site of drug metabolism and detoxification. More than 80% of liver blood flow comes from the gastrointestinal tract[4]. Hence, the liver is susceptible to anti-carcinogen toxicity. In many circumstances, when treatment with paclitaxel is preceded by cancer pain in advanced tumors, paclitaxel may be administered with fentanyl, which used to alleviate moderate to severe pain via intravenous, transdermal or mucosal routes[5,6]. However, it is unclear what role fentanyl plays in the side effects of paclitaxel.

Chronic anticancer therapy often induces ABCB1 expression in cancer cells and increases cellular efflux of anti-carcinogens. ABCB1, which is one of the most important members in the ABC transporter superfamily, can pump substrate drugs out of the cells against a concentration gradient with the use of energy from ATP hydrolysis[7,8]. The ABCB1 and CYP3A families share some substrates or modulators, including paclitaxel, cyclosporin A, verapamil, and imidazole antimycotic agents such as ketoconazole [9–11]. CYP3A4 is the most prevalent member of the CYP family of enzymes in the liver and is involved in the oxidation of a vast array of chemically unrelated drugs from almost every drug class [12,13]. Numerous studies have demonstrated that clinically relevant drug-drug interactions (DDIs) may occur at the metabolism level and are largely mediated by ABCB1 and CYP3A4 when an ABCB1 inhibitor is combined with a CYP3A4 substrate[14]. Previous studies also showed that fentanyl is a substrate of ABCB1, acting as an ABCB1 inhibitor[15,16]. Thus, alteration of ABCB1 transportation and CYP3A4 activity likely has effects on the pharmacokinetics of paclitaxel.

Herein, we hypothesize the effect of fentanyl on the hepatotoxicity of paclitaxel and offer some suggestions for clinical practice.

## Materials and Methods

### Cells and Animals

Cell lines were cultured in DMEM or RPMI-1640, supplemented with 10% FBS, at 37°C in a humidified atmosphere of 5% CO_2_. The human oral epidermoid carcinoma cell line, KB, and its vincristine-selected ABCB1-overexpressing derivative, KBv200, were gifts from Dr. Xu-Yi Liu (Cancer Hospital of Beijing, Beijing, China)[17]. The human breast carcinoma cell line, MCF-7, and its doxorubicin-selected ABCB1-overexpressing derivative MCF-7/adr; the human leukemia cell line, HL60, and its doxorubicin-selected ABCC1-overexpressing derivative, HL60/adr; and the human colon carcinoma cell line, S1, and its mitoxantrone-selected ABCG2-overexpressing derivative, S1-M1-80, were obtained from Dr. S.E.Bates (National Cancer Institute, National Institutes of Health, Bethesda, MD)[18–20].

KunMing male mice (7–8 weeks old, 28–38 g) were provided by the Center of Experimental Animals, Sun Yat-Sen University. Mice were housed in standard cages under a 12 h light-dark cycle at 22°C and 45–50% relative humidity and supplied with rodent chow and water for one week before use.

### Ethics Statement

This study was carried out in strict accordance with the recommendations in the Guide for the Care and Use of Laboratory Animals of the National Institutes of Health. All experiments were approved by the Committee on the Ethics of Animal Experiments of the Center of Experimental Animals, Sun Yat-Sen University, China. All experiments were performed under 2–3% isoflurane anesthesia in O_2_, and all efforts were made to minimize suffering. Mice had free access to sterilized food and water before they were euthanized by decapitation with anesthesia after blood or liver sample collection. No mice became ill during the experiments.

### *In-vivo* Pharmacokinetic Study

Mice were randomly divided into two groups: (1) paclitaxel (i.v., 18 mg/kg); (2) paclitaxel (i.v., 18 mg/kg) + fentanyl (i.v., 100 μg/kg). Treatments were administered through the caudal vein [21,22]. Five blood samples from each of five mice were collected from the retro-orbital plexus after the mice were anesthetized with 2% isoflurane at 0, 5, 15, 30, 45, 60, 90, 120, 240, 480, and 720 minutes post dosing of each group and placed in heparinized tubes. Blood samples were centrifuged at 4000 rpm for 10 min, and 400 μl plasma was removed and stored at -20°C until analysis.

Mice were randomly allocated into four groups: (1) paclitaxel (i.v., 18 mg/kg); (2) paclitaxel (i.v., 18 mg/kg) + fentanyl (i.v., 25 μg/kg); (3) paclitaxel (i.v., 18 mg/kg) + fentanyl (i.v., 50 μg/kg); (4) paclitaxel (i.v., 18 mg/kg) + fentanyl (i.v., 100 μg/kg). Five liver samples were collected after mice were anesthetized with 2% isoflurane at 1, 1.5, 2.5 and 10 h after the injection of each group. Liver samples were wiped with filter paper, rinsed with saline, dried to remove excess fluid and weighed. Each visceral sample was mixed with saline solution (4 times the weight of the sample), homogenized using a blender and stored at -20°C[23,24].

The SPE-column activation, sample extraction and the first two washes were performed by gravity. 4 μl internal standard solution (0.5 mg/ml docetaxel) was added to 400 μl plasma or tissue homogenate and vortex-mixed for 30 s. Subsequently, the full volumn of sample was pipetted onto the activated SPE-columns. After the second wash, columns were washed with acetonitrile, and the elution samples were collected and transferred to a clean tube and dried under nitrogen stream in the water bath at 40°C. The residues were dissolved with a 400 μl solution of 0.01 M NH_4_Ac (pH 5.0)-Ethanol (50:50, v/v), vortex-mixed for 5 min, and centrifuged at 10000 rpm for 5 min. The supernatant was transferred to a clean tube, and 50 μl was injected into the RP-HPLC system for analysis[21,25].

Paclitaxel concentration was analyzed by the RP-HPLC method on the Agilent 1100 system (Agilent Technologies, USA) as described previously[21]. The Waters Nova-park C_18_ (3.9×150 mm, 4 μm) chromatographic column was used for separation. The mobile phase was composed of acetonitrile-water (42:58, v/v) at a flow rate of 1.0 ml/min. The column temperature was maintained at 25°C. The detection wavelength was set at 227 nm. Non-compartmental pharmacokinetic parameters were calculated using WinNonlin (Version 5.0). The terminal-phase rate constant (λz) was calculated as the negative slope of the log-linear terminal portion of the serum concentration–time curve using linear regression with at least four last concentration–time points. The terminal-phase half-life (t_1/2_) was calculated as 0.693/λz. The area under the plasma concentration-time curve from zero to 12 hour (AUC_0-12_) was calculated by the linear trapezoidal rule for ascending data points. The maximum serum concentration (C_max_) was determined directly. The total area under the curve (AUC_0–∞_) was calculated as AUC_0-12_ + Ct/λz, where Ct is the last measurable concentration. The apparent volume of distribution associated with the terminal phase (Vz) was calculated as Vz = CL/λz, and the apparent total body clearance (CL) was calculated as CL = dose/AUC.

### Biological and Histological Study

Mice were randomly allocated into six groups (n = 5): (1) NaCl 0.9% (i.v.); (2) fentanyl (i.v., 100 μg/kg); (3) paclitaxel (i.v., 18 mg/kg); (4) paclitaxel (i.v., 18 mg/kg) + fentanyl (i.v., 25 μg/kg); (5) paclitaxel (i.v., 18 mg/kg) + fentanyl (i.v., 50 μg/kg); (6) paclitaxel (i.v., 18 mg/kg) + fentanyl (i.v., 100 μg/kg).Treatments were delivered through the caudal vein for 3 consecutive days. Blood samples were drawn from the retro-orbital plexus 72 h after administration of 2% isoflurane. AST and ALT were measured enzymatically using the corresponding kits. Mice were sacrificed, and the livers were embedded in paraffin blocks, sliced into 5 μm sections and placed onto glass slides. After hematoxylin-eosin (HE) staining, the slides were observed, and photos were taken using an optical microscope. The examining pathologist was blinded to the identity and analysis of the pathology slides.

### Assays of CYP3A4 Activity

The effect of fentanyl on CYP3A4 activity in insect membranes (V4820, Promega Corporation, Madison, WI), human hepatic microsomes and (HC-0015, Lifeline Cell Technology, Suite Z–Frederick, MD) and mice hepatic microsomes (M1000, XenoTech. LLC, Lenexa, KS) ectopically expressing human CYP3A4 was determined using 96-well microtiter plates according to the Promega protocol. The activity of CYP3A4 was examined using the P450-GloTM CYP3A4 Assay (Luciferin-IPA) according to the protocol (V9001, Promega, USA).

### Assays of ABCB1 ATPase Activity

The fentanyl-stimulated ABCB1 ATPase activity was estimated using the P-gp-GloTM assay system (V3601, Promega)[26].

### Doxorubicin and Rhodamine 123 Accumulation

Flow cytometry was used as described previously to examine intracellular accumulation of doxorubicin and rhodamine 123 in various cells [27]. Verapamil, a known ABCB1 inhibitor, was used as a positive control[28].

### Analysis of ABCB1 Expression

Western blot analysis, RT-PCR and Real-time quantitative PCR were performed as described previously to identify whether fentanyl affects ABCB1 protein expression [27]. The level of ABCB1 mRNA was expressed as a ratio relative to the GAPDH mRNA in each sample. Relative quantification of ABCB1 was performed using the threshold cycle difference method[29].

### Cytotoxicity Assay

The MTT assay to assess the cytotoxicity of anti-carcinogens in cells was performed as described previously [27].

### Statistical Analysis

Statistical analysis of the quantitative data generated in the experiments, which were repeated at least three times, was performed using the SPSS statistical software package (version 17.0, SPSS Inc., Chicago, IL, USA). Data were analyzed by two-tailed Student's *t*-test and considered significant if *P* < 0.05.

## Results

### Effect of Fentanyl on the Pharmacokinetics of Paclitaxel in Mice

**Fig 1:**
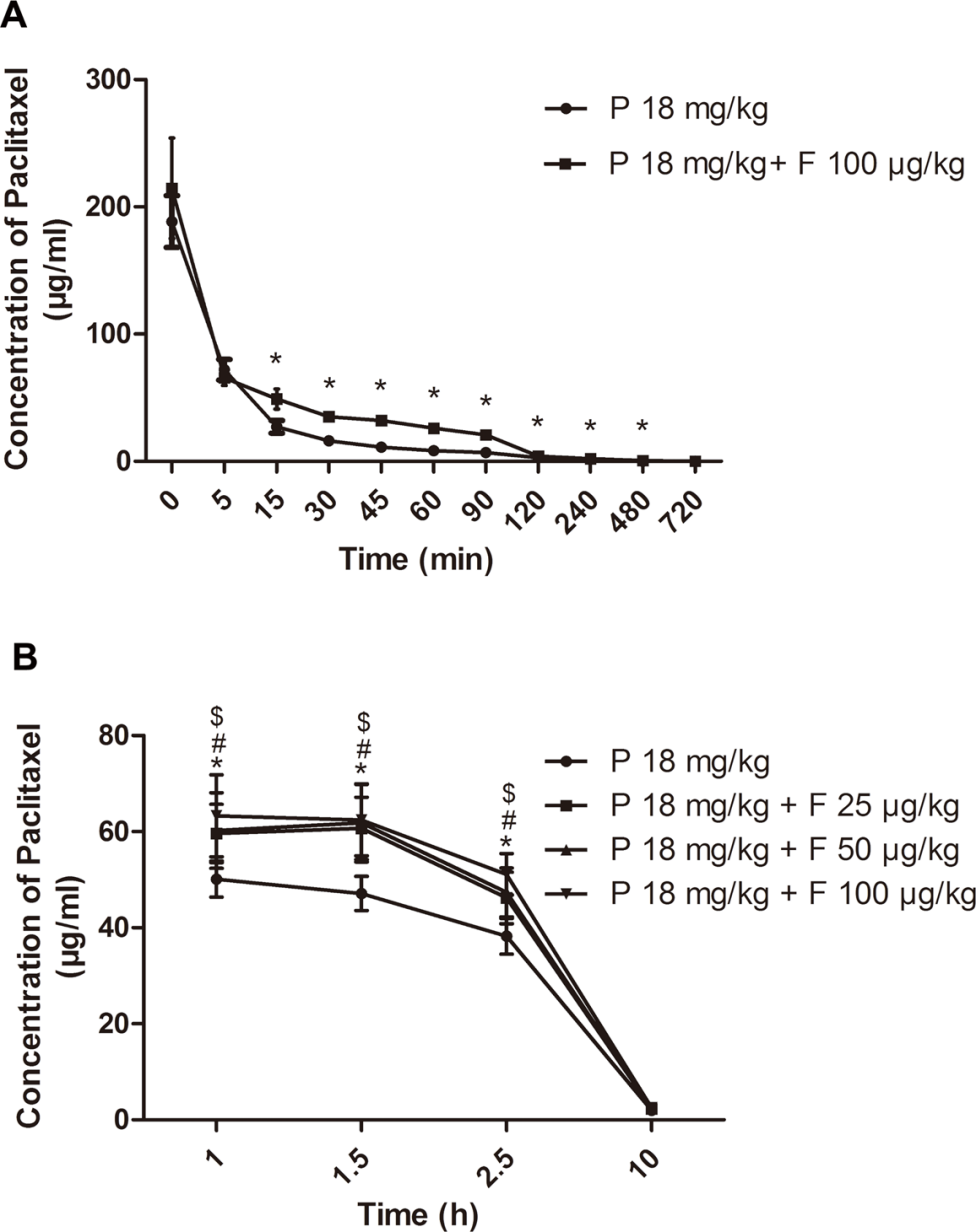
Pharmacokinetic and liver distribution profile of paclitaxel after different treatments. (A) Plasma concentration–time profiles of paclitaxel. (B) Liver distribution concentration–time profiles of paclitaxel. Data are expressed as mean±SD, n = 5. P = paclitaxel; F = fentanyl. Paclitaxel concentrations of combination groups were significantly higher than that of paclitaxel-only group (*P < 0.05, ^#^ *P* < 0.05, ^$^ *P* < 0.05).

The RP-HPLC method employed in our experiment is applicable as the standard curve demonstrates fine linearity (R^2^>0.999), recovery and precision (data not shown). The mean plasma concentration-time profiles and the main pharmacokinetic parameters after different treatments are presented (Fig 1A and Table 1). The AUC of paclitaxel with fentanyl was significantly increased, the t_1/2_ was prolonged from 1.455 hours to 2.344 hours, and the CL was decreased from 10.997 ml/h to 7.014 ml/h, displaying an increased accumulation and decreased clearance of paclitaxel after the combination. These findings indicated that fentanyl could enhance the systemic exposure of paclitaxel and thereby increase the likelihood of hepatotoxicity.

**Table 1:** Pharmacokinetic parameters of paclitaxel in plasma between groups combining without- and with- fentanyl.

Parameters	with Saline	with Fentanyl	*P* value
C_max_ (ng/ml)	188.471 ± 20.402	214.611 ± 39.531	0.366
t_1/2_ (h)	1.455 ± 0.197	2.344 ± 0.357*	0.019
AUC_0-12_ (ng·h/ml)	48.973 ± 7.428	77.901 ± 7.879*	0.01
AUC_0-∞_ (ng·h/ml)	49.103 ± 7.938	78.414 ± 5.598*	0.006
Vd (ml)	23.08 ± 4.308	23.719 ± 4.143	0.862
CL (ml/h)	10.997 ± 1.562	7.014 ± 1.135*	0.023

AUC_0-12_ = area under the plasma concentration-time curve from zero to 12 hour; AUC_0-∞_ = area under the plasma concentration-time curve from zero to infinity; C_max_ = peak plasma concentration; t_1/2_ = elimination half-time; Vd = volume of distribution; CL = total clearance. Values are expressed as mean±SD, n = 5.

*P < 0.05 compared with control group.

### Effect of Fentanyl on the Liver Distribution of Paclitaxel in Mice

The liver concentrations of paclitaxel in mice are presented (Fig 1B). The concentrations of paclitaxel in the three combination groups were all higher than those of the paclitaxel-only group at 1 h, 1.5 h and 2.5 h, and the results were dependent of fentanyl dose. There was no significant difference in the concentrations of paclitaxel among the four groups at 10 h. These data indicated that fentanyl can result in the increased liver distribution of paclitaxel.

### Biological and Histological analysis

**Fig 2:**
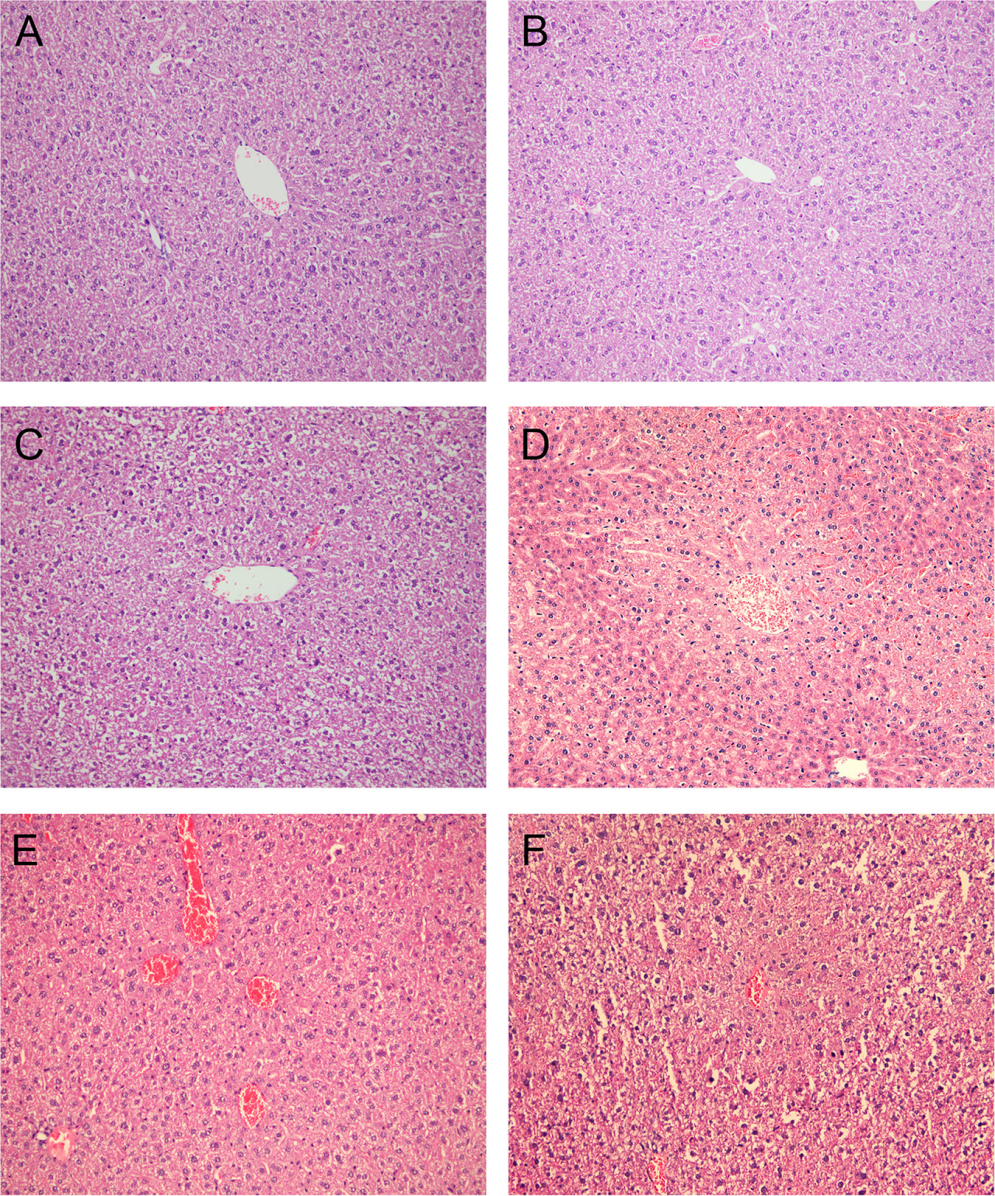
Histological change of livers in mice after different treatments. Representative photographs (magnification ×200) of livers in mice from different groups: (A) Equivalent volumes of saline as paclitaxel, (B) fentanyl 100 μg/kg, (C) paclitaxel 18 mg/kg, (D) paclitaxel 18 mg/kg + fentanyl 25 μg/kg, (E) paclitaxel 18 mg/kg + fentanyl 50 μg/kg, (F) paclitaxel 18 mg/kg + fentanyl 100 μg/kg.

To confirm the hepatotoxicity of paclitaxel-fentanyl administration, we measured AST and ALT and examined the hepatic lobules under optical microscopy. ALT, the hepatic-specific marker, was markedly elevated in groups treated with paclitaxel compared with the control group (Table 2). ALT increased dramatically to 88.2±13.9 U/L upon treatment with paclitaxel 18 mg/kg + fentanyl 100 μg/kg, which is more than 2-fold the level detected in the control group. This result suggests impaired liver function in paclitaxel-treated mice, especially when paclitaxel is combined with high doses fentanyl. No obvious morphological alteration was found in the control group or fentanyl-only group. However, mild congestion was observed in the central veins in the group treated with paclitaxel 18 mg/kg (Fig 2). The congestion in the central veins of the paclitaxel 18 mg/kg + fentanyl 25 μg/kg treatment group was more severe than that of the group treated with paclitaxel 18 mg/kg only, and significant hydropic and fatty degeneration were observed in hepatocytes. Local necrosis of peripheral hepatocytes around the central veins was observed in the group treated with paclitaxel 18 mg/kg + fentanyl 100 μg/kg. Given the biological and histological results, we concluded that the co-administration could produce severe toxicity to the liver.

**Table 2:** Alterations of ALT and AST in mice among groups after different treatments.

Groups	ALT (U/L) (*P* Value)	AST (U/L) (*P* Value)
Saline	36.1±8.83	86.6±8.36
F 100μg/kg	38.7±10.22 (0.755)	82.1±6.76 (0.508)
P 18mg	60.5±8.75* (0.027)	87.8±10.46(0.884)
P 18mg + F 25μg/kg	60.1±4.86* (0.015)	93.4±6.89 (0.338)
P 18mg + F 50μg/kg	60.3±11.45* (0.044)	85.4±6.84 (0.857)
P 18mg + F 100μg/kg	88.2±13.9** (0.005)	85.8±7.29 (0.907)

After different treatments for three consecutive days, ALT (aspartate transaminase) and AST (alanine aminotransferase) were measured. P = paclitaxel; F = fentanyl. Values are expressed as mean±SD, n = 5.

*P < 0.05 and

**P < 0.01 compared with saline group.

### Effects of Fentanyl on various Cells

Fentanyl cytotoxicity in different cell lines was examined using the MTT assay. More than 85% of the cells were viable when concentrations of fentanyl reached 20 μM in the cell lines used for the study. We selected 10 μM as the maximal concentration for the MTT assay in case the medium in the plate was not sufficient. Fentanyl 10 μM significantly enhanced the cytotoxicity of doxorubicin in KBv200 and MCF-7/adr cells by 10.9- and 7.5-fold, respectively, and paclitaxel enhanced the cytotoxicity by 5.17- and 5.08-fold, respectively (Table 3). However, these effects were not observed in KB or MCF-7 cells. Fentanyl did not affect the cytotoxicity of doxorubicin in HL60/adr or S1-M1-80 cells, nor did it affect the cytotoxicity of paclitaxel in HL60/adr or S1-M1-80 cells. Moreover, fentanyl did not distinctly sensitize any cells to cisplatin, which is not a substrate of ABCB1, ABCC1 or ABCG2. These results suggest that fentanyl significantly enhanced the cytotoxicity of ABCB1 substrates (doxorubicin and paclitaxel) in ABCB1-overexpressing cells.

**Table 3:** Effect of fentanyl on enhancing drug cytotoxicity in ABCB1-, ABCC1- and ABCG2-overexpressing cell lines.

Compounds	IC_50_ ± SD (μM) (fold-enhancement)	
	KB	KBv200 (ABCB1)
Doxorubicin	0.623±0.008 (1.00)	12.651±0.045 (1.00)
+ 2.5 μM Fentanyl	0.615±0.012 (1.01)	2.720±0.035** (4.65)
+ 5.0 μM Fentanyl	0.618±0.005 (1.01)	1.560±0.018** (8.11)
+ 10 μM Fentanyl	0.615±0.011 (1.01)	1.158±0.012** (10.9)
+ 10 μM Verapamil	0.632±0.013 (0.99)	0.629±0.083** (20.1)
Paclitaxel	0.021±0.003 (1.00)	3.841±0.087 (1.00)
+ 2.5 μM Fentanyl	0.022±0.005 (0.95)	1.850±0.085** (2.08)
+ 5.0 μM Fentanyl	0.021±0.002 (1.00)	1.661±0.106** (2.31)
+ 10 μM Fentanyl	0.021±0.006 (1.00)	0.743±0.084** (5.17)
+ 10 μM Verapamil	0.021±0.004 (1.00)	0.382±0.027** (10.1)
Cisplatin	0.328±0.013 (1.00)	2.368±0.079 (1.00)
+ 10 μM Fentanyl	0.346±0.022 (0.95)	2.317±0.157 (1.02)
+ 10 μM Verapamil	0.322±0.017 (1.02)	2.334±0.186 (1.01)
	MCF-7	MCF-7/adr (ABCB1)
Doxorubicin	0.405±0.008 (1.00)	9.803±0.971 (1.00)
+ 2.5 μM Fentanyl	0.398±0.025 (1.02)	2.213±0.223** (4.43)
+ 5.0 μM Fentanyl	0.412±0.021 (0.98)	1.735±0.103** (5.65)
+ 10 μM Fentanyl	0.409±0.019 (0.99)	1.307±0.052** (7.50)
+ 10 μM Verapamil	0.404±0.020 (1.00)	1.243±0.091** (7.89)
Paclitaxel	0.084±0.004 (1.00)	4.593±0.030 (1.00)
+ 2.5 μM Fentanyl	0.087±0.013 (0.97)	1.739±0.036** (2.64)
+ 5.0 μM Fentanyl	0.092±0.003 (0.91)	1.267±0.027** (3.63)
+ 10 μM Fentanyl	0.084±0.005 (1.00)	0.905±0.081** (5.08)
+ 10 μM Verapamil	0.084±0.010 (1.00)	0.431±0.006** (10.7)
Cisplatin	8.853±0.426 (1.00)	11.266±0.580 (1.00)
+ 10 μM Fentanyl	8.359±0.426 (1.06)	11.773±1.389 (0.96)
+ 10 μM Verapamil	9.023±0.562 (0.98)	12.013±1.213 (0.94)
	HL60	HL60/adr (ABCC1)
Doxorubicin	0.135±0.013 (1.00)	4.203±0.247 (1.00)
+ 2.5 μM Fentanyl	0.131±0.021 (1.03)	4.550±0.156 (0.92)
+ 5.0 μM Fentanyl	0.142±0.024 (0.95)	3.981±0.231 (1.06)
+ 10 μM Fentanyl	0.143±0.034 (0.94)	4.012±0.192 (1.05)
+ 50 μM MK571	0.133±0.016 (1.02)	0.943±0.051** (4.46)
	S1	S1-M1-80 (ABCG2)
Topotecan	0.855±0.018 (1.00)	9.523±0.117 (1.00)
+ 2.5 μM Fentanyl	0.898±0.035 (0.95)	9.213±0.083 (1.03)
+ 5.0 μM Fentanyl	0.912±0.041 (0.94)	8.985±0.103 (1.06)
+ 10 μM Fentanyl	0.909±0.019 (0.94)	9.187±0.102 (1.04)
+ 0.5 μM FTC	0.834±0.012 (1.03)	0.774±0.050** (12.3)

Cell survival was determined by MTT. The fold-enhancement of drug cytotoxicity was calculated by dividing the IC_50_ value for cells with the anti-carcinogens in the absence of inhibitor by that obtained in the presence of inhibitor. Values are expressed as mean±SD, n = 3.

**P < 0.01 compared with that obtained in the absence of inhibitor. Verapamil, MK571 and FTC are positive control.

### Fentanyl increased the Accumulation of Doxorubicin and Rhodamine 123 in ABCB1-overexpressing Cells

**Fig 3:**
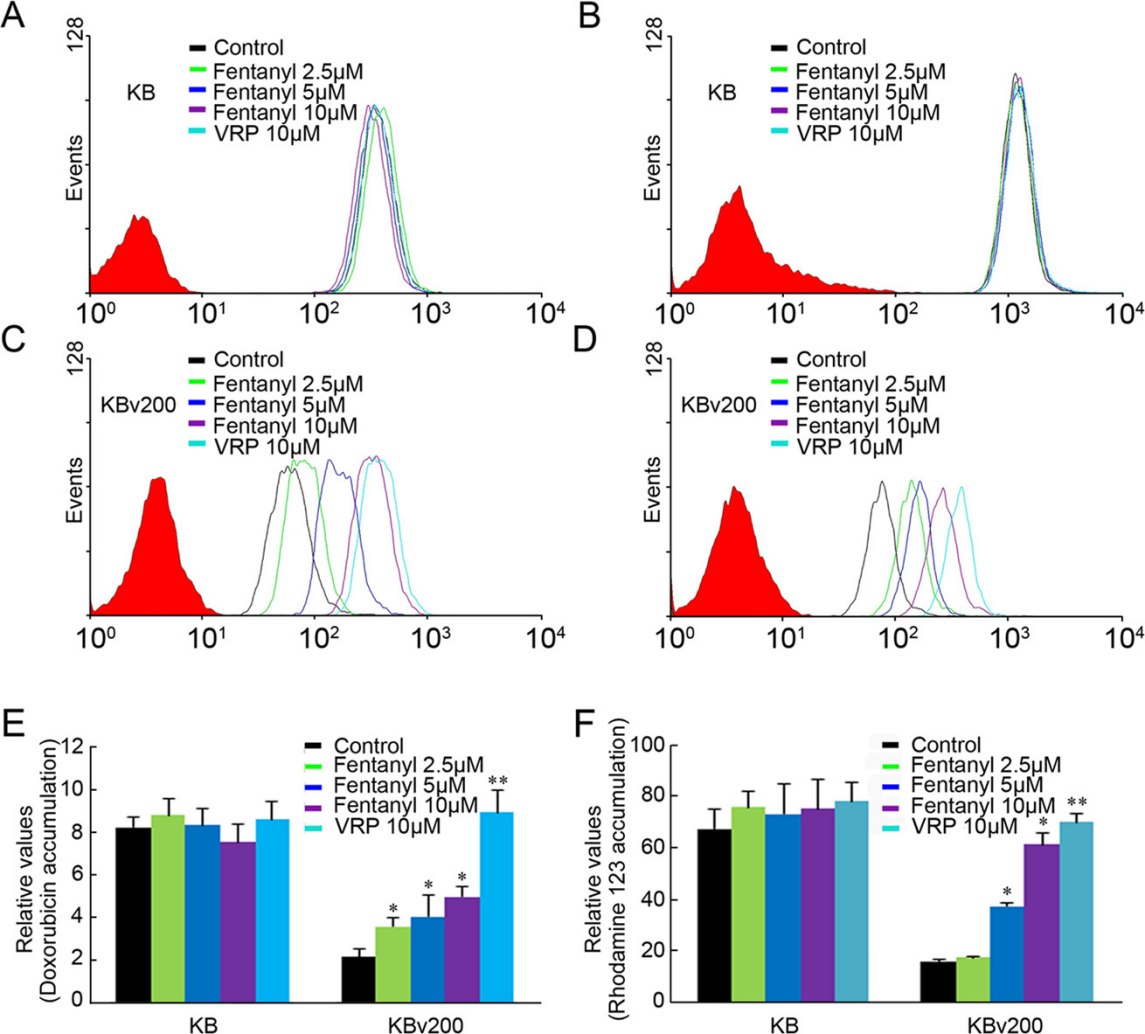
Effect of fentanyl on the accumulation of doxorubicin and rhodamine123 in KB and KBv200 cells. The accumulation of doxorubicin (A, C) and rhodamine 123 (B, D) were measured by flow cytometric analysis. VRP = verapamil, as a positive control. Data from (E) (F) are shown as mean±SD, n = 3. *P < 0.05, **P< 0.01 *versu*s control group.

**Fig 4:**
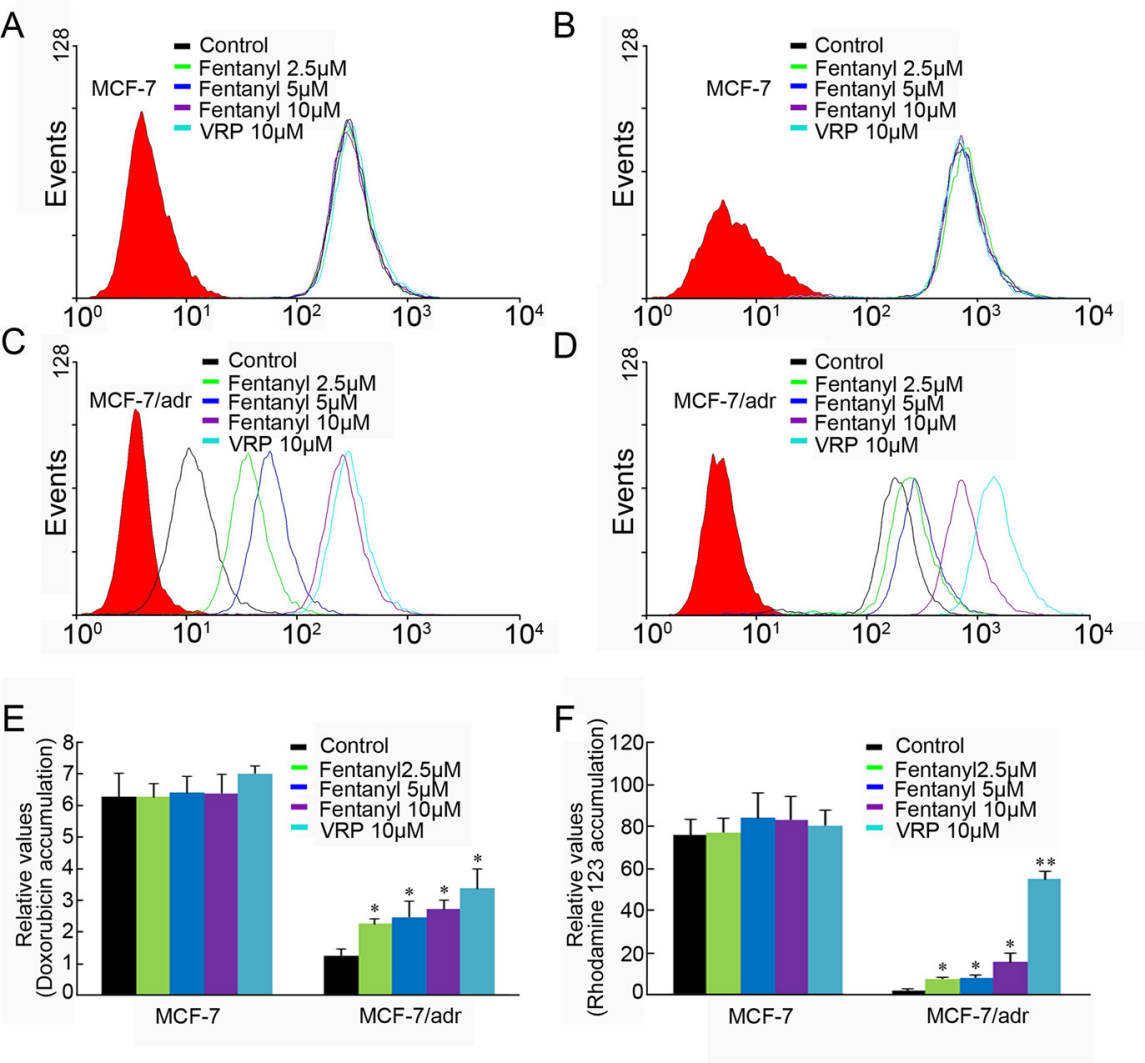
Effect of fentanyl on the accumulation of doxorubicin and rhodamine123 in MCF-7 and MCF-7/adr cells. The accumulation of doxorubicin (A, C) and rhodamine 123 (B, D) were measured by flow cytometric analysis. VRP = verapamil, as a positive control. Data from (E) (F) are shown as mean ± SD, n = 3.Columns, mean relative values of control group; bars, SD. *P < 0.05, **P< 0.01 *versu*s control group.

The above results indicate that fentanyl can enhance the cytotoxicity of anti-carcinogens in ABCB1-overexpressing cells. To ascertain whether this effect was related to ABCB1, we examined the effect of fentanyl on the accumulation of doxorubicin and rhodamine123 in ABCB1-overexpressing KBv200 and MCF-7/adr cells as well as their parental cell lines. Fentanyl did not affect the accumulation of doxorubicin or rhodamine 123 in the two parental sensitive cell lines. In the absence of fentanyl, the intracellular levels of doxorubicin and rhodamine 123 were low in KBv200 and MCF-7/adr cells. However, fentanyl significantly increased the intracellular levels of doxorubicin and rhodamine 123 in a concentration-dependent manner in KBv200 and MCF-7/adr cells (Figs 3 and 4). The fluorescence index of doxorubicin in the presence of 2.5, 5 and 10 μM fentanyl was increased by 1.66-, 1.89- and 2.32-fold, respectively, in KBv200 cells. Furthermore, the index of rhodamine 123 in the presence of 5 and 10 μM fentanyl was increased by 2.35- and 3.85-fold, respectively, in KBv200 cells. Fentanyl 2.5, 5.0 and 10.0 μmol/L increased the intracellular accumulation of doxorubicin and rhodamine123 by 1.83-, 2.01-, 2.21-fold and 3.53-, 3.76-, 7.17-fold, respectively, in MCF-7/adr cells.

### Fentanyl did not Alter ABCB1 Expression

**Fig 5:**
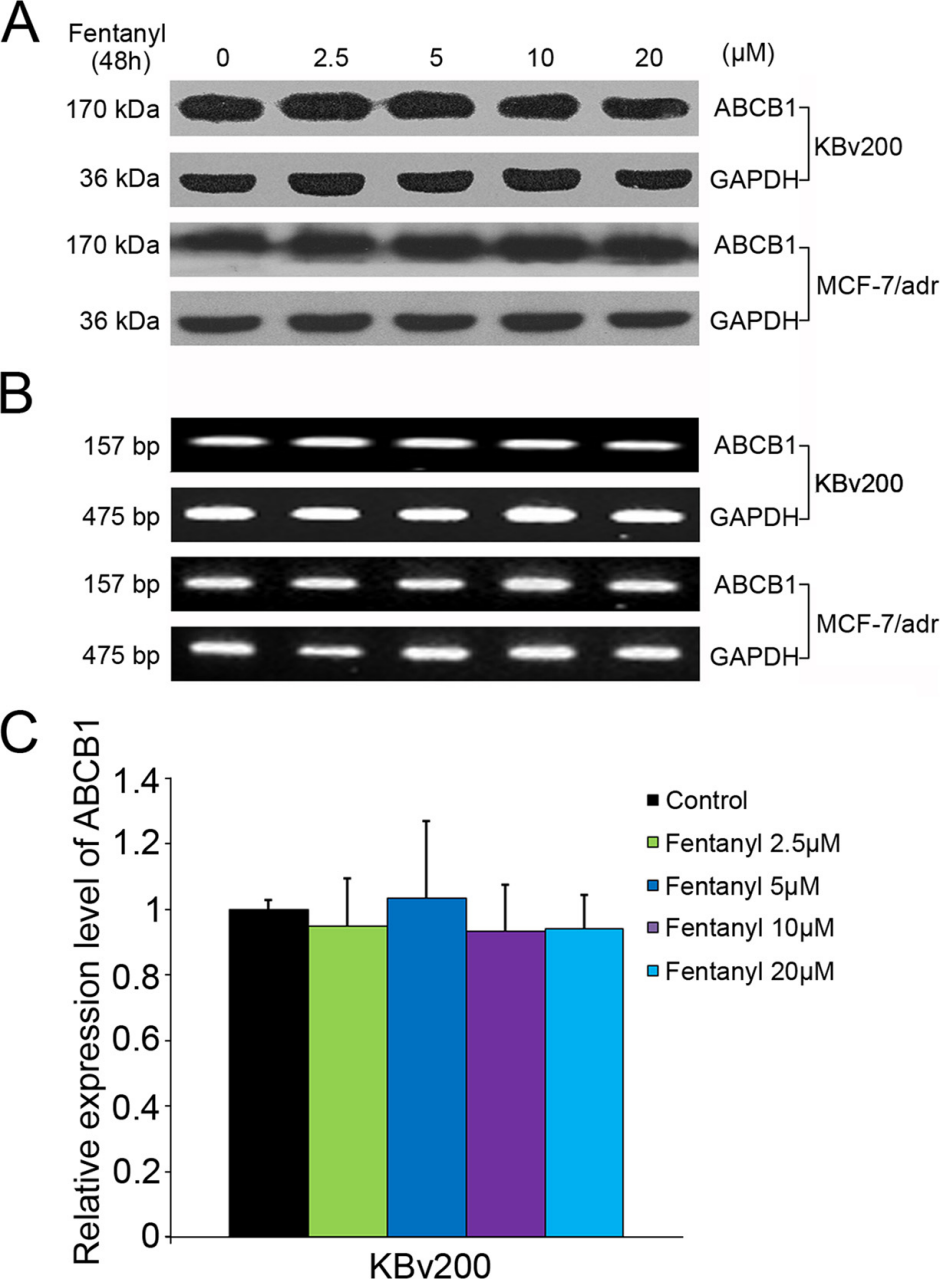
Effect of fentanyl on the expression of ABCB1 in ABCB1-overexpressing cell lines. Expression of ABCB1 was measured from: (A) equal amounts of total cell lysates were loaded and detected by Western blot; (B) the mRNA level of ABCB1 was determined by RT-PCR; (C) summary data from the mRNA level of ABCB1 determined by RT real-time-PCR. Data are expressed as mean ± SD, n = 3. There were no differences among different groups.

According to previous research, either inhibiting the function or decreasing the expression of ABCB1 can sensitize ABCB1-overexpressing cells to substrate anti-carcinogens. Therefore, we examined the effect of fentanyl on the protein and mRNA levels of ABCB1. We found that the expression of ABCB1 protein or mRNA was not significantly altered by fentanyl (Fig 5).

### Effect of Fentanyl on the Activity of Hepatic CYP3A4 and ABCB1 ATPase

**Fig 6:**
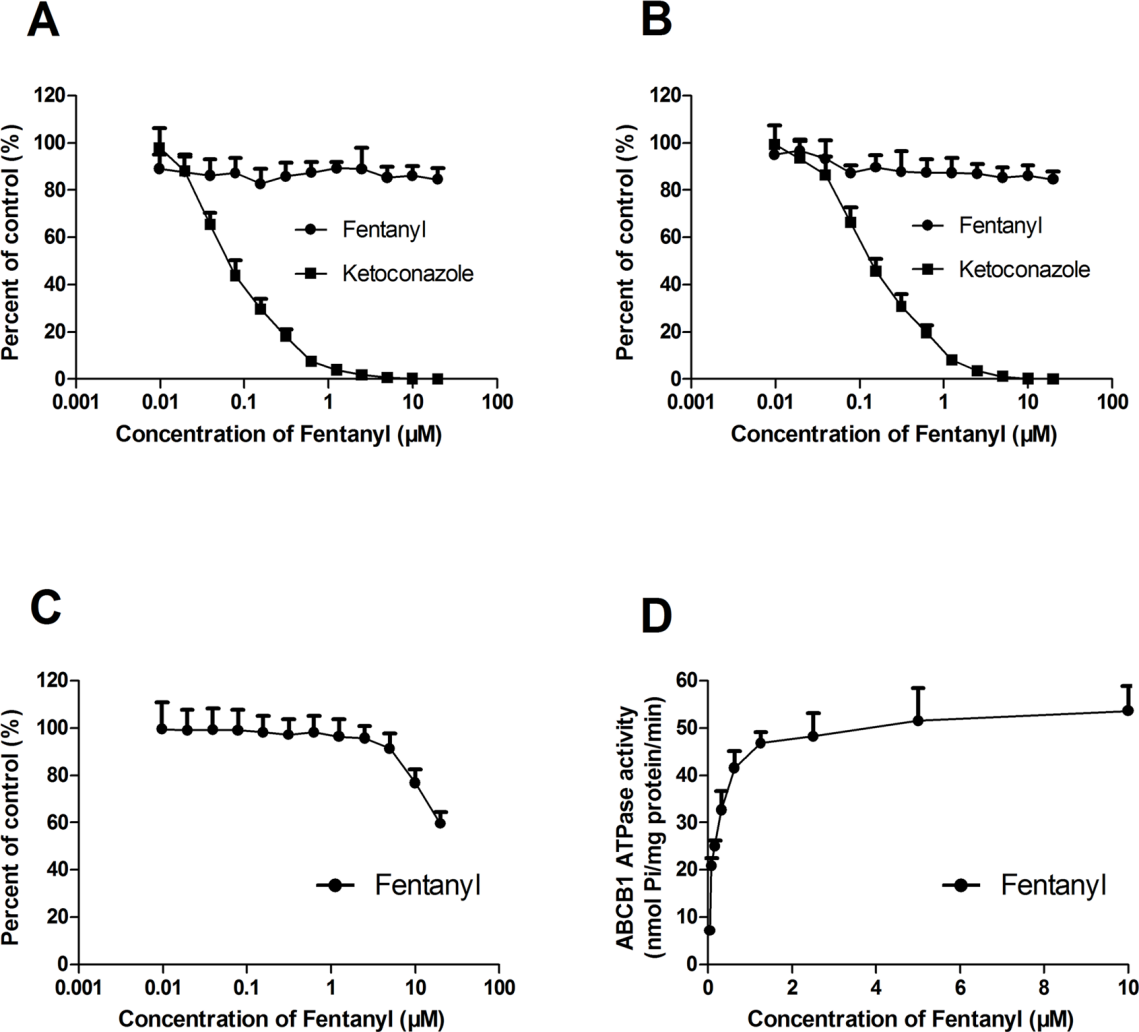
Effect of fentanyl on the activity of hepatic CYP3A4 and ABCB1 ATPase. Activity of hepatic CYP3A4 was examined from: (A) human CYP3A4 ectopic expressing insect membranes, (B) human hepatic microsomes and (C) mice hepatic microsomes. Activity of (A, B) was inhibited by less than 20%, and the activity of (C) was significantly inhibited over 40% in present of 20 μM fentanyl with three wells of ketoconazole as positive control inhibited almost 95% of activity of hepatic microsome (C) (data not shown). ABCB1 ATPase assays (D) were performed according to the instruction of Pgp-Glo™ Assay Systems. Data are shown as mean±SD, n = 3.

To determine whether fentanyl can affect the activity of CYP3A4, we determined its effect on human and mouse liver microsomes as well as insect membranes ectopically expressing human CYP3A4 *in vitro*. Ketoconazole significantly inhibited the activity of CYP3A4 in a concentration-dependent manner. We observed that fentanyl significantly inhibited CYP3A4 activity in mice by more than 40% at a concentration of 20 μM (Fig 6C), but these effects were not seen in humans (Fig 6A and 6B). To evaluate the effect of fentanyl on the ATPase activity of ABCB1, we assessed ABCB1-mediated ATP hydrolysis with different concentrations of fentanyl (Fig 6D). We concluded from the results that fentanyl activated the ATPase activity of ABCB1 in a concentration-dependent manner.

## Discussion

Fentanyl is often prescribed concomitantly for pain control in advanced tumor patients being treated with chemotherapy. However, the influence of fentanyl on the hepatotoxicity of paclitaxel remains to be elucidated.

In the present study, we demonstrated for the first time that the pharmacokinetics of paclitaxel in mice were significantly altered by fentanyl. The concentrations of paclitaxel in the paclitaxel-fentanyl group were higher than those of the paclitaxel-only group. The AUC of paclitaxel with fentanyl were significantly increased, implying increased accumulation. Paclitaxel exhibited lower CL and longer t_1/2_ when it was co-administered with fentanyl, and we preliminarily speculated that the clearance of paclitaxel through the hepatic or renal route was depressed by fentanyl.

However, we observed that the distribution of paclitaxel in the liver was increased in a dose-dependent manner when paclitaxel was combined with fentanyl. To investigate this potential conflict, we tested the liver function and found increased ALT and pathological damage in the combination groups, suggesting that liver impairment in paclitaxel-treated mice was due to the prolonged duration and increased distribution of paclitaxel in the liver secondary to fentanyl co-administration, especially at high doses.

CYP enzyme inhibition and the resulting change in drug plasma levels are the causes of many clinically important DDIs [30,31]. Here, we observed that fentanyl significantly inhibited the activity of CYP3A4 in mouse liver microsomes, indicating that fentanyl may reduce the metabolism of paclitaxel via inhibition of CYP3A4, resulting in increased paclitaxel accumulation. However, fentanyl did not inhibit the activity of pure human hepatic CYP3A4 membranes or human liver microsomes. We speculate that there are possible species-specific differences in the interaction between CYP3A4 and fentanyl.

There is a large overlap in the affinities of various substrates of the ABCB1 and CYP3A isozymes, making it difficult to identify whether the observed *in vivo* interaction is a result of CYP3A or ABCB1 inhibition or both. In the present study, we demonstrated that fentanyl significantly potentiated the cytotoxicity of established ABCB1 substrate anti-carcinogens and increased the accumulation of doxorubicin and rhodamine 123 in ABCB1-overexpressing cells. However, fentanyl did not significantly alter the cytotoxicity of cisplatin, which is a non-ABCB1 substrate, in either sensitive or resistant cells. Furthermore, fentanyl did not significantly change the sensitivity of doxorubicin in ABCC1-overexpressing cells or topotecan in ABCG2-overexpressing cells. These findings suggest that cytotoxicity enhancement in resistant cells by fentanyl is specific to ABCB1. The concentrations of fentanyl used in our experiments were 2.5, 5.0 and 10.0 μM, which are higher than the clinical concentrations used in patients. However, higher concentrations may be obtained in tumor tissues than in normal tissues and plasma due to the impaired tumor vasculature[32]. Therefore, the concentrations of fentanyl used in our *in vitro* assays might be achieved in tumor tissues during chemotherapy.

The overexpression of ABCB1 is generally known to mediate multidrug resistance (MDR) by actively pumping its substrates out of cells [7]. We observed that fentanyl did not affect the expression of ABCB1 at either the protein or mRNA level. However, similar to the cytotoxicity data, fentanyl was found to significantly increase the intracellular accumulation of doxorubicin and rhodamine 123 in ABCB1-overexpressing cells in a dose-dependent manner. Moreover, fentanyl increased the ATPase activity of ABCB1 in a dose-dependent manner. Because energy derived from ATP hydrolysis is needed for ABC transporters to pump their substrate drugs out of cells, the profile of drug-stimulated ATPase activity in the ABCB1-overexpressing membrane is thought to embody the natural interactions between the ABC transporters and the substrates[33]. Taking our data into account, fentanyl may inhibit ABCB1’s efflux function by increasing its ATPase activity, resulting in increased intracellular concentration of its substrate anti-carcinogens, and fentanyl is likely to be a substrate and competitive inhibitor of ABCB1. Our results are consistent with previous research[15].

Many reports have demonstrated that ABCB1 can mediate DDIs when two ABCB1 substrates compete for the same binding site or when an ABCB1 substrate is combined with an ABCB1 inhibitor [34,35]. These reports indicate that the tissue distribution and therapeutic efficacy of paclitaxel may be affected when paclitaxel is combined with fentanyl. Furthermore, in the liver and kidneys, ABCB1 increases the rate of drug removal from circulation by increasing drug excretion into the bile and urine. Therefore, the inhibition of ABCB1 can result in changes in the level of drug absorption and excretion, leading to increased plasma concentrations. Inhibition of ABCB1 also impairs its protective function in normal cells, as in the case of paclitaxel treatment, which results in increased hepatotoxicity[36,37]. However, there are possible species-specific differences in the substrate specificities of ABCB1 and CYP enzymes[38,39]. Clinicians should understand the consequences of ABCB1-mediated absorption and excretion and CYP-dependent metabolism on the pharmacokinetics of paclitaxel, and they should be aware that fentanyl may alter the function of these systems. Thus, much attention should be paid to the decreased dose or extended interval of exposure paclitaxel when it is co-administered with fentanyl.

However, *in vivo* studies in other species, including humans, are needed to truly understand the significance of ABCB1 and CYP3A4 in determining the disposition and elimination of paclitaxel when combined with fentanyl.

In summary, biological and histological analysis in our study showed that fentanyl significantly enhanced the hepatotoxicity caused by paclitaxel when the drugs were administered together in mice. We demonstrated that fentanyl could enhance hepatotoxicity by increasing the accumulation of paclitaxel through the inhibition of CYP3A4 and ABCB1 transport activity. These findings provided a rationale for issuing a recommendation to avoid the accumulation-induced hepatotoxicity of paclitaxel when it is co-administered with fentanyl.

## Supporting Information

S1 Fig The cytotoxicity of fentanyl alone in various cell lines. (TIF)

## Abbreviations

ABC: ATP-binding cassette
ABCB1: ABC transporter-subfamily B member 1
ABCC1: ABC transporter-subfamily C member 1
ABCG2: ABC transporter-subfamily G member 2
RP-HPLC: reversed phase high-performance liquid chromatography
AST: aspartate transaminase
ALT: alanine aminotransferase
MTT: 1-(4, 5-dimethylthiazol-2-yl)-3,5-diphenylformazan
SPE: solid phase extraction
AUC_0-12_: area under the plasma concentration-time curve from zero to 12 hour
AUC_0-∞_: area under the plasma concentration-time curve from zero to infinity
C_max_: peak plasma concentration
t_1/2_: elimination half-time
Vd: volume of distribution
CL: total clearance
MDR: multidrug resistance
TACE: transcatheter hepatic artery chemotherapy embolization
VRP: verapamil
FTC: fumitremorgin C
