# Supplementary figures and images for: Fentanyl Enhances Hepatotoxicity of Paclitaxel via Inhibition of CYP3A4 and ABCB1 Transport Activity in Mice
Source: PLoS One. 2015 Dec 3;10(12):e0143701. doi: 10.1371/journal.pone.0143701 (PMC4669130; doi:10.1371/journal.pone.0143701)

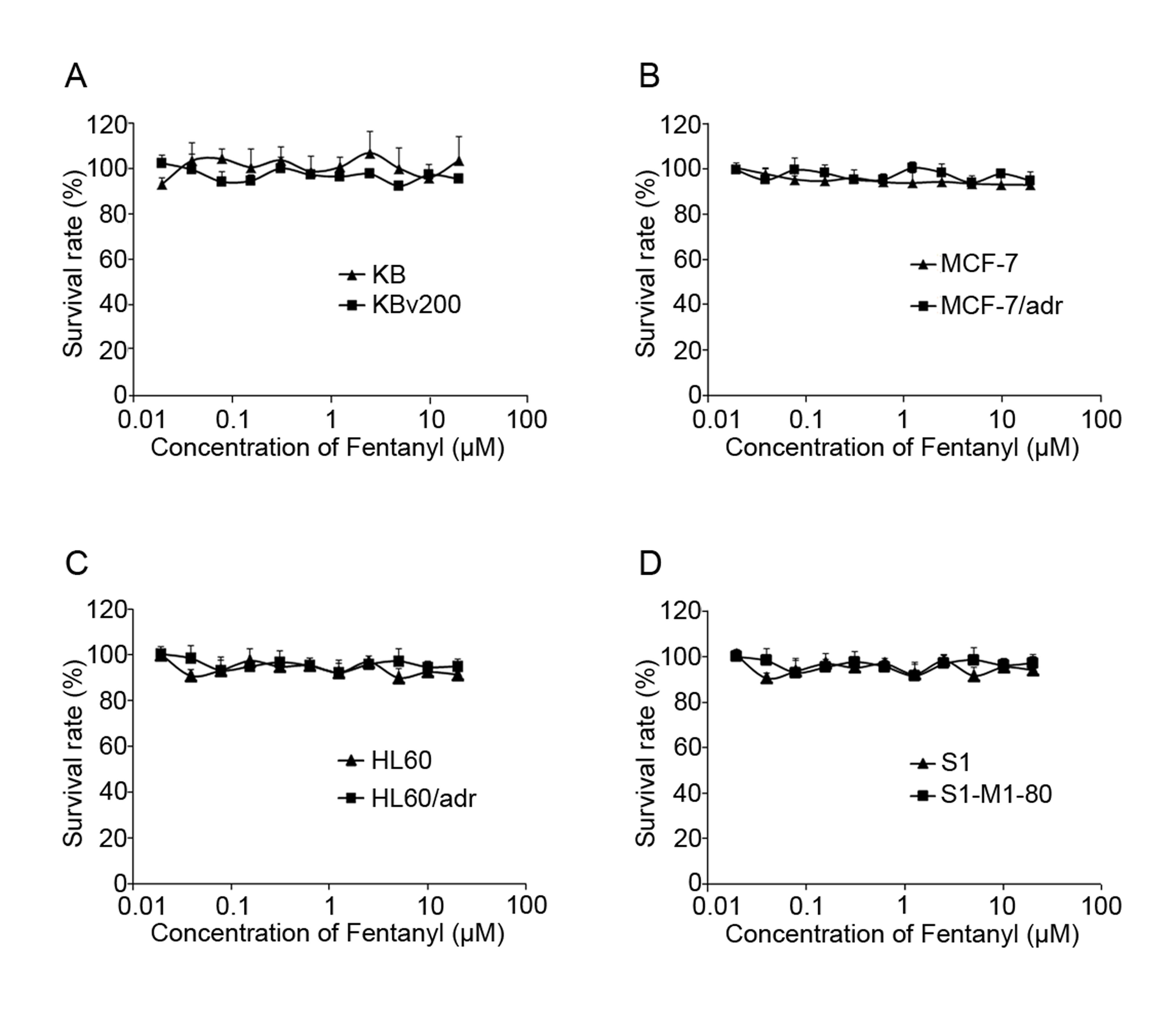

Supplement: S1 Fig — (TIF) [file pone.0143701.s001.tif]
